# Aging-dependent loss of GAP junction proteins Cx46 and Cx50 in the fiber cells of human and mouse lenses accounts for the diminished coupling conductance

**DOI:** 10.18632/aging.203247

**Published:** 2021-07-04

**Authors:** Xiao-Dong Gong, Yan Wang, Xue-Bin Hu, Shu-Yu Zheng, Jia-Ling Fu, Qian Nie, Ling Wang, Min Hou, Jia-Wen Xiang, Yuan Xiao, Qian Gao, Yue-Yue Bai, Yi-Zhi Liu, David Wan-Cheng Li

**Affiliations:** 1 The State Key Laboratory of Ophthalmology, Zhongshan Ophthalmic Center, Sun Yat-Sen University, Guangzhou, Guangdong 510230, China

**Keywords:** gap junctions, connexins, lens, cataract, oxidative stress

## Abstract

The homeostasis of the ocular lens is maintained by a microcirculation system propagated through gap junction channels. It is well established that the intercellular communications of the lens become deteriorative during aging. However, the molecular basis for this change in human lenses has not been well defined. Here, we present evidence to show that over 90% of Cx46 and Cx50 are lost in the fiber cells of normal human lenses aged 50 and above. From transparent to cataractous lenses, while Cx43 was upregulated, both Cx46 and Cx50 were significantly down-regulated in the lens epithelia. During aging of mouse lenses, Cx43 remained unchanged, but both Cx46 and Cx50 were significantly downregulated. Under oxidative stress treatment, mouse lenses develop *in vitro* cataractogenesis. Associated with this process, Cx43 was significantly upregulated, in contrast, Cx46 and Cx50 were sharply downregulated. Together, our results for the first time reveal that downregulation in Cx46 and Cx50 levels appears to be the major reason for the diminished coupling conductance, and the aging-dependent loss of Cx46 and Cx50 promotes senile cataractogenesis.

## INTRODUCTION

The ocular lens is a unique organ that lacks both vascular and nerve systems [1, 2]. Its homeostasis is maintained by a microcirculation system propagated through gap junction channels [3, 4]. The lens gap junction consists of three different connexin proteins, α1 (Cx43), α3 (Cx46), and α8 (Cx50), which are encoded by three genes: *Gja1*, *Gja3,* and *Gja8,* respectively [5]. Earlier studies showed that Cx43 is mainly expressed in lens epithelial cells [6]. In contrast, Cx46 is dominantly expressed in lens fiber cells, and Cx50 is expressed in both lens epithelial and fiber cells [7].

Structurally, connexin proteins contain four transmembrane domains with both termini located inside the cells. This transmembrane property leaves two extracellular loops and one intracellular loop [8]. Six connexin protein subunits become oligomerized into a homomeric connexon (if all the subunits are the same) or a heteromeric connexon (if two and more subunits are present) [9]. A connexon is also called a hemichannel, and two hemichannels can assemble into a full channel through the interactions of the extracellular loops [10].

Both hemichannels and full gap junction channels play an important role in maintaining lens transparency [3, 11–25]. These gap junction channels are capable of transporting small molecular nutrients and metabolites [23–25]. More recently, it was found that these channels are also responsible for the delivery of antioxidants [25].

Cataract is defined as any opacity in the lens [26]. It is derived from either genetical mutations (known as congenital cataracts) [27] or environment stress conditions (inducing non-congenital cataracts) [28]. Previous studies have revealed that mutations in either *Gja3* or *Gja8* cause various types of congenital cataract [29–31]. Depending on the genetic background, homozygous and heterozygous mutations may display different phenotypes in lens pathology [32–33]. Gene knockout studies confirm the earlier mutation results. More importantly, such studies help to explore the mechanisms mediating lens pathogenesis. It was found that *Gja3* (-/-) knockout mice grew normally but presented with classic nuclear cataract [34]. This is derived from elevated intracellular calcium level, activating proteases and causing protein degradation [34–35]. On the other hand, *Gja8* (-/-) knockout mice displayed microphthalmia and zonular pulverulent cataract [36], which is derived from reduced proliferation of lens epithelial cells, and delayed lens fiber cell maturation [37].

For non-congenital cataracts, oxidative stress has been suggested as an initiating factor [38–40]. We previously demonstrated that stress factors including oxidative stress and other stress conditions, can activate apoptosis of lens epithelial cells, which causes subsequent cataractogenesis [41–48]. Senile cataracts develop as the lens ages. During this process, various molecular changes have been reported from different laboratories. These changes include DNA damage [49], protein oxidation [50–53], loss of enzyme activity [51], truncations of gap junction proteins at defined sites [19–20], and other changes [53–55].

It is well established that the intercellular communications of the lens become deteriorative during aging. However, the molecular basis for this change has not been well defined in human lenses. In the present study, we utilized automated Wes [48, 56] and examined the expression patterns of the three gap junction proteins (Cx43, Cx46, and Cx50) in transparent human lenses and the capsular epithelia from individual cataract patients of different age groups from 50-year old to 87-year old. Our results showed that from transparent lenses to cataractous lenses, the three gap junction proteins display contrast change patterns. While Cx43 is upregulated, both Cx46 and Cx50 are downregulated in the lens epithelial cells. In the fiber cells of normal human lenses aged 50 and old, more than 90% downregulation in Cx46 and Cx50 is detected. In the aging mice, we also found that while Cx43 remains relatively stable, both Cx46 and Cx50 displayed age-dependent downregulation. Since oxidative stress is implicated in senile cataract development [38–40], we therefore also examined the changes of Cx43, Cx46, and Cx50 in mouse lenses treated with glucose oxidase (GO), an enzyme that generates hydrogen peroxide during *in vitro* lens organ culture [48]. It was found that oxidative stress induces upregulation of Cx43 but causes significant downregulation of both Cx46 and Cx50. Together, our results for the first time reveal that downregulation of Cx46 and Cx50 appears to be the main reason for the diminished coupling conductance of the aged lens besides the age-dependent truncations at the defined sites [19–20], and that the aging-dependent loss of Cx46 and Cx50 promotes lens pathology, senile cataractogenesis.

## RESULTS

### Expression of Cx43 in different age groups of human cataract patients

We recently analyzed the expression patterns of both sumoylation ligases and desumoylation enzymes and their target, Pax6 in different groups of cataract patients. Our results revealed that the expression pattern changes in some of these molecules can serve as a molecular signature for senile and complicated cataracts [48]. Because the gap junction proteins play a fundamental role in lens transparency, we examined whether their expression patterns were altered when comparing transparent lenses with cataract lenses and in cataract lenses of different ages. To obtain the answer to the above question, we examined the protein levels of three different types of gap junction proteins in capsular epithelia isolated from four pairs of normal human lenses (one 45-year-old female, and three male individuals, aged 61, 64 and 65; Supplementary Table 1) and 48 cataract patients of different ages [12 patients aged 50–59 (Supplementary Table 2), denoted “50s”; 12 patients aged 60–69 (Supplementary Table 3), denoted “60s”; 12 patients aged 70–79 (Supplementary Table 4), denoted “70s”; and 12 patients aged 80–90 (Supplementary Table 5), denoted “80s”].

**Figure 1:**
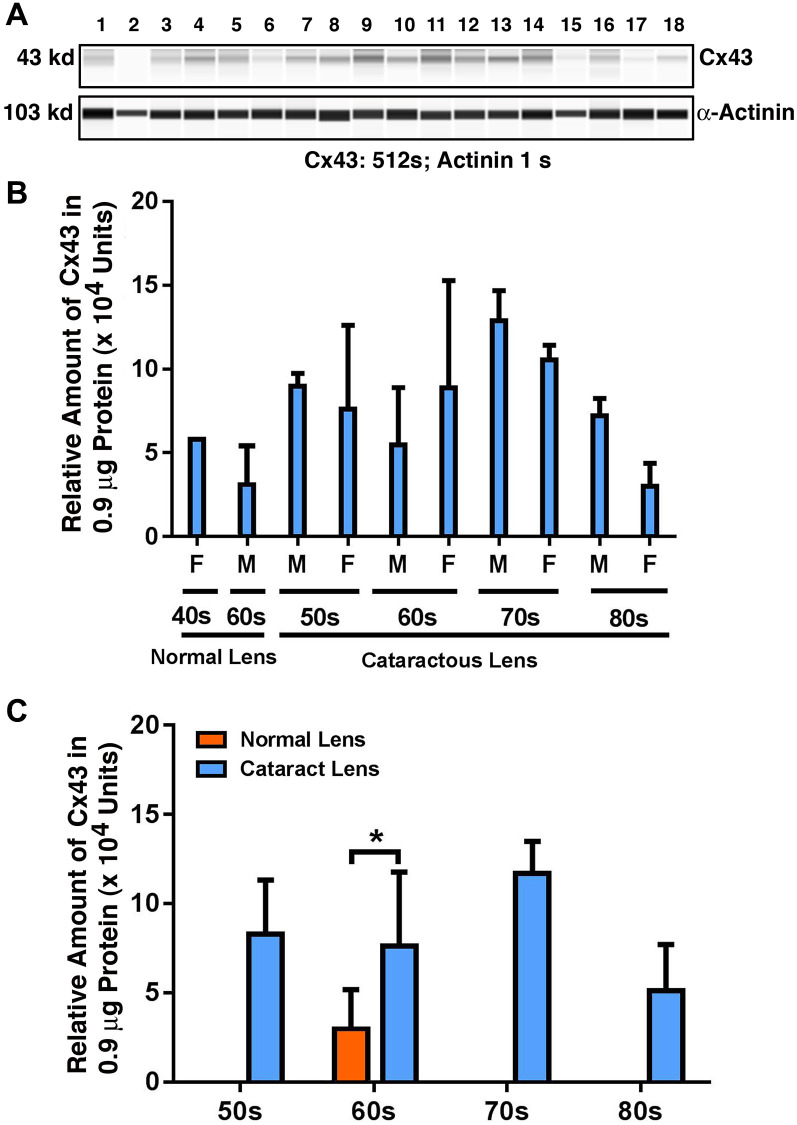
**The automated western immunoblot (AWI) analyses of connexin 43 (Cx43) in human normal and cataractous lenses of different age groups.** AWI was performed on a Wes (ProteinSimple) as described recently [48, 56]. Briefly, each sample was loaded with 0.9 μg total protein and then analyzed with the Size Separation Master Kit and Split Buffer (12–230 kDa) according to the manufacturer’s standard instruction using anti-Cx43 antibody (for antibody information, see Experimental Procedures) with a dilution factor of 1:100. The Campass software (Protein Simple, version 4.1.5) was used to program the PeggySue-robot and for presentation (**A**) and quantification (**B**–**C**). Output western blot style data (**A**) were displayed with exposure time indicated, and the quantification data (**B**–**C**) were displayed from the software-calculated average of seven exposures (1–512s). (**B**) Quantification results show gender difference. Each bar represents an average of 8 samples for cataract lenses but one sample for normal human lens of 40s and three samples for normal human lenses of 60s. Lanes 1–2 represent normal lenses, and lanes 3–18 represent cataractous lenses of different age groups (3–6, 50s; 7–10, 60s; 11–14, 70s and 15–18, 80s). (**C**) Quantification results show age difference. ^*^p < 0.05.

As shown in Figure 1A and 1B, in normal lenses from 40-year-old to 60-year-old donors, the protein level for Cx43 was downregulated. However, when comparing transparent lenses of normal human to cataractous lenses from patients of similar ages, Cx43 was upregulated about 50% (Figure 1C). Among the cataract patients of different age groups, the Cx43 remained relatively stable from 50s to 60s, and became upregulated about 35% from 60s to 70s. From 70s to 80s, however, Cx43 was significantly downregulated nearly 50%. Differences between males and females were detectable from 70s to 80s, with males appearing to have higher levels of Cx43 than females (Figure 1C).

### Expression of Cx46 in human cataract patients from different age groups

**Figure 2:**
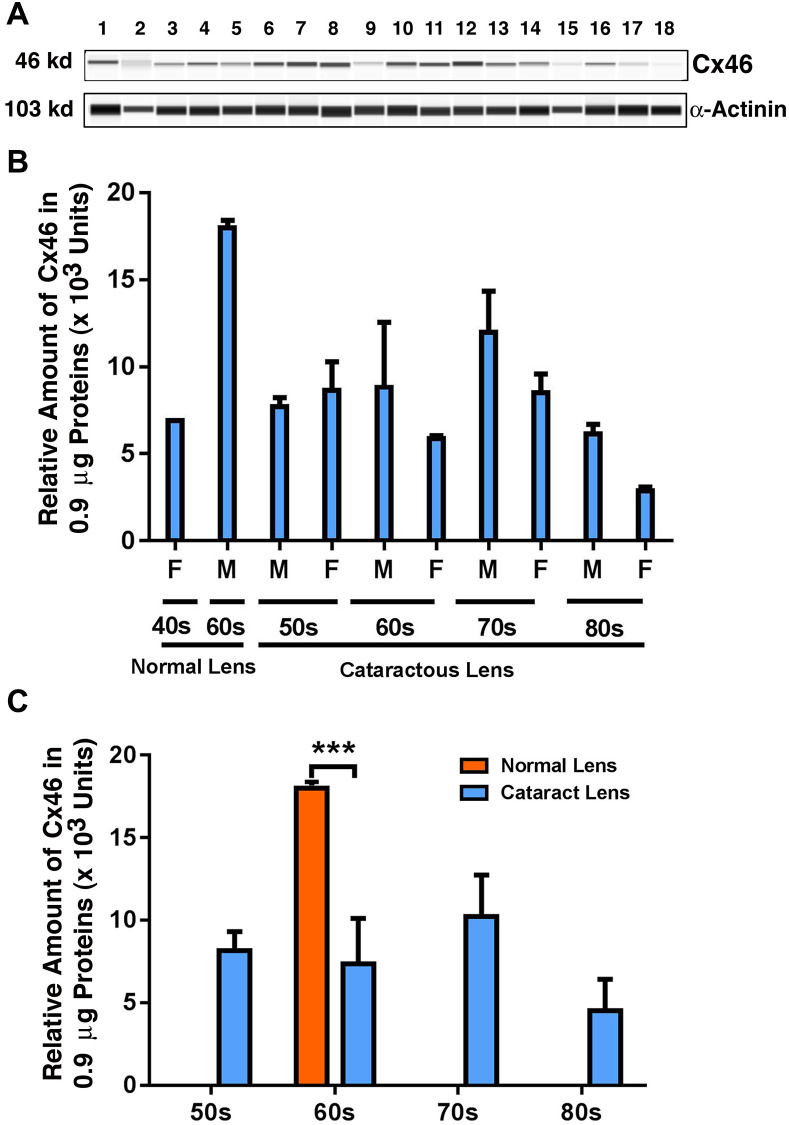
**The automated western immunoblot (AWI) analyses of connexin 46 (Cx46) in normal and cataractous lenses of different age groups.** AWI was performed on a Wes (ProteinSimple) as described recently [48, 56]. Briefly, each sample was loaded with 0.9 μg total protein and then analyzed with the Size Separation Master Kit and Split Buffer (12–230 kDa) according to the manufacturer’s standard instruction using anti-Cx46 antibody (for antibody information, see Experimental Procedures) with a dilution factor of 1:20. The Campass software (Protein Simple, version 4.1.5) was used to program the PeggySue-robot and for presentation (**A**) and quantification (**B**–**C**). Output western blot style data (**A**) were displayed with exposure time indicated in Figure 1, and the quantification data (**B**–**C**) were displayed from the software-calculated average of seven exposures (1–512s). (**B**) Quantification results show gender difference. Each bar represents an average of 8 samples for cataract lenses but one sample for normal human lens of 40s and three samples for normal human lenses of 60s. Lanes 1–2 represent normal lenses, and lanes 3–18 represent cataractous lenses of different age groups (3–6, 50s; 7–10, 60s; 11–14, 70s and 15–18, 80s). (**C**) Quantification results show age difference. ^***^p < 0.001.

Next, we examined the possible changes in the expression pattern for Cx46: the connexin that demonstrated dominant expression in fiber cells in previous studies [7]. As shown in Figure 2A and 2B, in normal human lenses from 40-year-old to 60-year-old donors, the protein levels for Cx46 were upregulated by approximately 1.5-fold. Between transparent and cataract lenses of the similar age group (only available for the 60s group), the Cx46 was downregulated by over 50% (Figure 2C). Among the cataract patients of different age groups, the Cx46 also remained relatively stable from 50s to 60s. From 60s to 70s, Cx46 was upregulated by approximately 34%. From 70s to 80s, Cx46 was significantly downregulated by almost 50% (Figure 2C). Differences between males and females were detectable for Cx46 from the 60s group to the 80s group, with males appearing to have higher levels of Cx46 than females (Figure 2B).

### Expression of Cx50 in different age groups of human cataract patients

**Figure 3:**
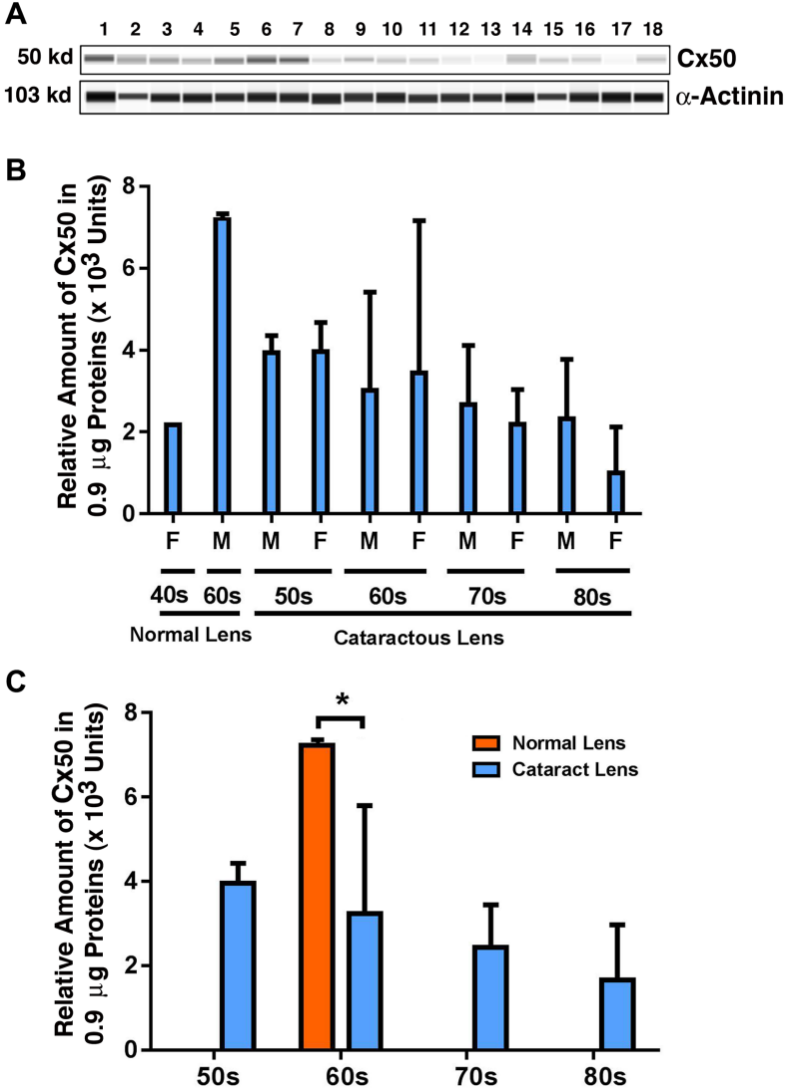
**The automated western immunoblot (AWI) analyses of connexin 50 (Cx50) in normal and cataractous lenses of different age groups.** AWI was performed on a Wes (ProteinSimple) as described recently [48, 56]. Briefly, each sample was loaded with 0.9 μg total protein and then analyzed with the Size Separation Master Kit and Split Buffer (12–230 kDa) according to the manufacturer’s standard instruction using anti-Cx50 antibody (for antibody information, see Experimental Procedures) with a dilution factor of 1:20. The Campass software (Protein Simple, version 4.1.5) was used to program the PeggySue-robot and for presentation (**A**) and quantification (**B**–**C**). Output western blot style data (**A**) were displayed with exposure time indicated in Figure 1, and the quantification data (**B**–**C**) were displayed from the software-calculated average of seven exposures (1–512s). (**B**) Quantification results show gender difference. Each bar represents an average of 8 samples for cataract lenses but one sample for normal human lens of 40s and three samples for normal human lenses of 60s. Lanes 1–2 represent normal lenses, and lanes 3–18 represent cataractous lenses of different age groups (3–6, 50s; 7–10, 60s; 11–14, 70s and 15–18, 80s). (**C**) Quantification results show age difference. ^*^p < 0.05.

Finally, we examined the possible changes in the expression pattern for Cx50: the connexin known to be expressed in both epithelial and fiber cells [7]. As shown in Figure 3A and 3B, from 40-year-old to 60-year-old normal lenses, the protein level for Cx50 was upregulated by approximately 2.5-fold. Among the cataract patients of different age groups, differently from Cx46, Cx50 was slightly downregulated from 50s to 60s, and this downregulation became more prominent, reaching about 18% from 60s to 70s (Figure 3C). From 70s to 80s, Cx50 was further downregulated by approximately 22%, becoming less than 50% of the Cx50 level in the 50s group (Figure 3C). Between transparent and cataract lenses of the similar age group (also only available for the 60s group), Cx50 was downregulated by over 50% (Figure 3C). Differences between males and females were also detectable for Cx50 from 70s to 80s, with males appearing to have a higher level of Cx50 than females (Figure 3B).

### The capsular epithelia of human lenses have far more Cx43 than Cx46 and Cx50

**Figure 4:**
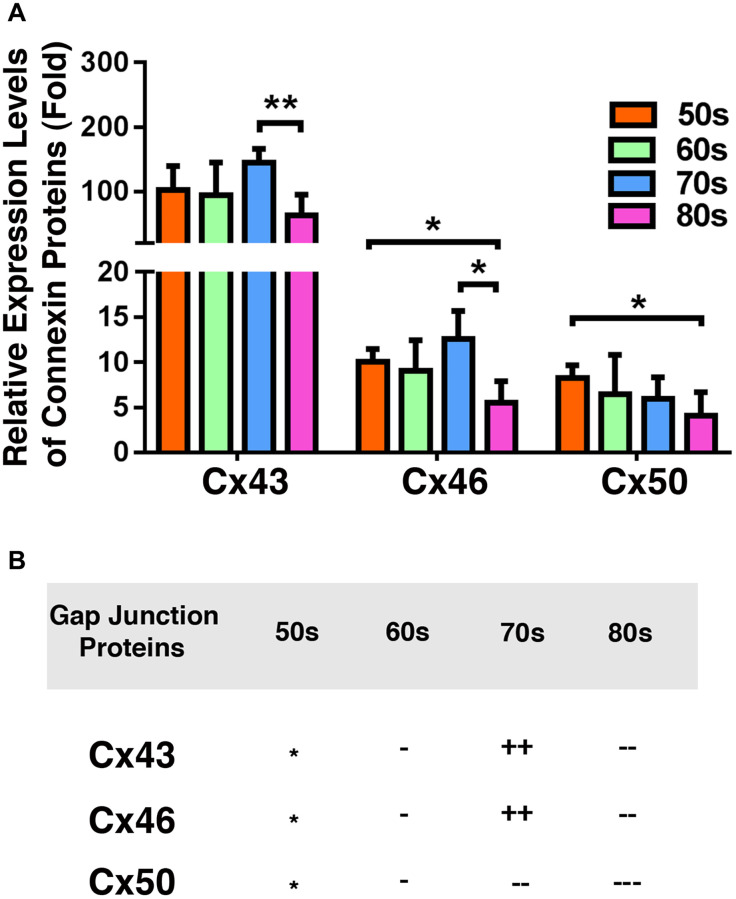
**Comparison of the connexins Cx43, Cx46 and Cx50 levels in the epithelia of human cataractous lenses of different age groups.** (**A**) Relative expression levels of Cx43, Cx46 and Cx50 in the epithelia of human cataractous lenses of different age groups as indicated (50s, 60s,70s, 80s). Each bar represents an average of 12 samples for cataract lenses. (**B**) Summary of age-dependent changes of Cx43, Cx46 and Cx50 in cataract samples of different age groups. The levels of all samples from the patients of 50s age group are used as references indicated by star symbol ‘^*^’. +, ++, and +++ represent increases in protein levels between 0.1% and 24.99%, 25% and 50%, and >50%, respectively; −, −−, and −−− stands for decreases in protein levels between 0.1% and 24.99%, 25% and 50%, and >50%, respectively.

We next compared the relative levels of Cx43, Cx46, and Cx50 in the epithelial samples of different age groups of cataract patients. As shown in Figure 4A, Cx43 exhibited the highest concentration in all patients of different age groups. The Cx43 level was almost 90% higher than that of Cx46, and more than 90% higher than that of Cx50 (Figure 4A). This was expected as Cx43 was previously found to be the main connexin in lens epithelial cells [7]. Interestingly, in the cataract patients of different age groups, Cx46 levels were slightly higher than Cx50 levels. Another feature of these three connexin proteins is that both Cx43 and Cx46 displayed some upregulation from 60s to 70s, and then became significantly downregulated from 70s to 80s (Figure 4B). By contrast, Cx50 became steadily downregulated from 50s to 80s. The downregulation was initially slow and became faster at older ages (Figure 4B). We were unable to collect the fiber cells from cataract patients. Therefore, we conducted studies on age-dependent changes in normal human lenses of different age groups.

### More than 90% of Cx46 and Cx50 is lost in the fiber cells of the normal human lenses aged 54 or older

**Figure 5:**
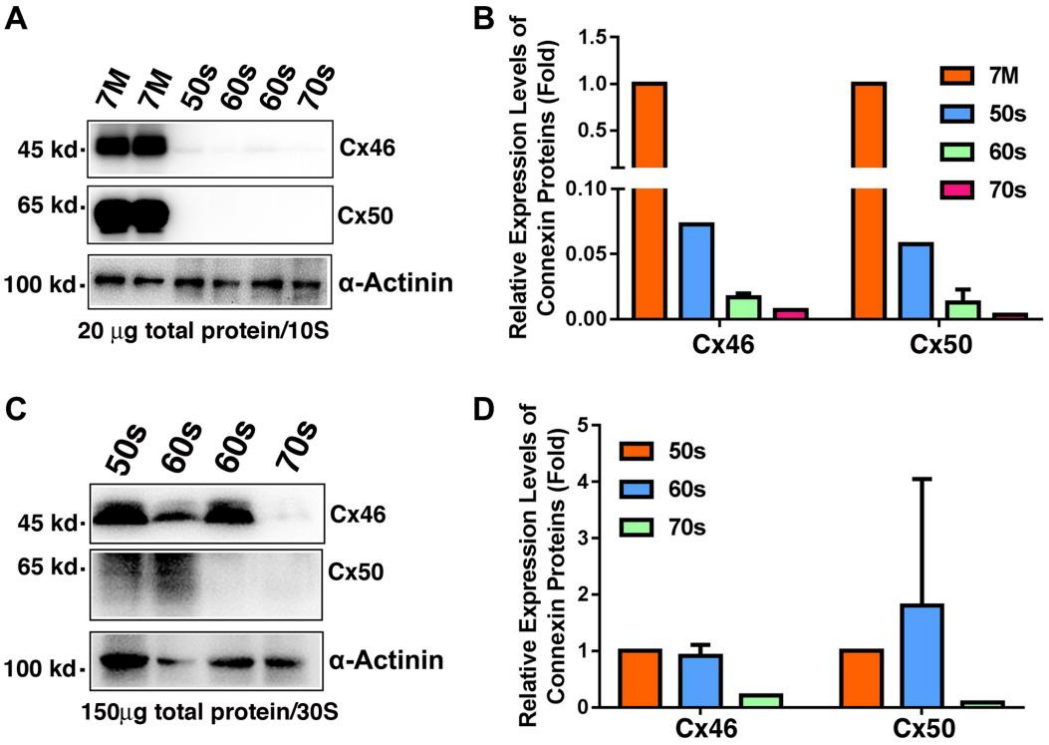
**Age-dependent changes of Cx46 and Cx50 in normal human lens fiber cells of different age groups as determined by regular western blot analysis.** (**A**) Western blot results of Cx46 and Cx50 in human lens fibers of different age groups. Note that in 20 μg of total proteins, Cx46 and Cx50 are abundantly expressed in the 7M human lens but become barely detectable in human lenses aged 54 and older. α-Actinin was showed as a loading control. (**B**) Quantification results show age-dependent changes of Cx46 and Cx50 in the fiber cells of different age groups as determined in (**A**). (**C**) Western blot results of Cx46 and Cx50 in human lens fibers of different age groups. Note that in 150 μg of total proteins, Cx46 is intact with relatively strong signal in human lenses of aged 65 or younger. Cx50 appears as degraded protein. Both Cx46 and Cx50 become undetectable after 60s. α-Actinin was showed as a loading control. (**D**) Quantification results show age-dependent changes of Cx46 and Cx50 in the fiber cells of different age groups as determined in (**C**).

Previous studies have shown that truncations of the gap junction proteins, especially truncations at the N-terminal domains and the cytoplasmic loop cause decrease in gap junction coupling [19]. These experiments have been done in four pairs of human lenses aged 19, 22, 55 and 62 [19] or exogenously expressed gap junction proteins Cx50 in *Xenopus* oocytes [20]. To further define the molecular basis for the age-dependent loss of the coupling conductance of the ocular lenses, we isolated total proteins from the fiber cells of normal human lenses from 7M to 74-year old donors (Supplementary Table 1) for regular western blot analysis. We utilized the antibodies from Santa Cruz Biotechnology, and the Cx46 antibody (sc-365394) recognizes the fragment of the residues 301 to 435, and the Cx50 antibody (sc-373801) identifies the fragment of the residues 228 to 292. Since we focused on the changes of the total intact gap junction proteins Cx46 and Cx50 in lens fiber cells, we did not separate the fiber cells into different layers. As shown in Figure 5A, both Cx46 and Cx50 were easily detected in the fiber cells of 7M donor lens. Under the same experimental conditions (20 μg of total proteins were used, and the exposure time was 10 seconds), Cx46 was marginally detectable and Cx50 was undetectable in the donors of 50s to 70s. A quantitative analysis of the western blot results in Figure 5A revealed that more than 90% of Cx46 and Cx50 is lost in the fiber cells of the normal human lenses aged 54 and older (Figure 5B).

To better visualize the Cx46 and Cx50 signals in the fiber cells of normal human lenses aged 54 and older, we loaded 150 μg of total protein per sample for the next western blot analysis. As shown in Figure 5C, while intact Cx46 was detectable in lens fiber cells of normal human lenses younger than 60s, Cx50 appeared as the degraded protein in these fiber cells (Figure 5C). In the fiber cells of normal human lenses older than 70s, both Cx46 and Cx50 were undetectable (Figure 5D).

### Expression of Cx43, Cx46, and Cx50 in mouse lens epithelial cells of different age groups

**Figure 6:**
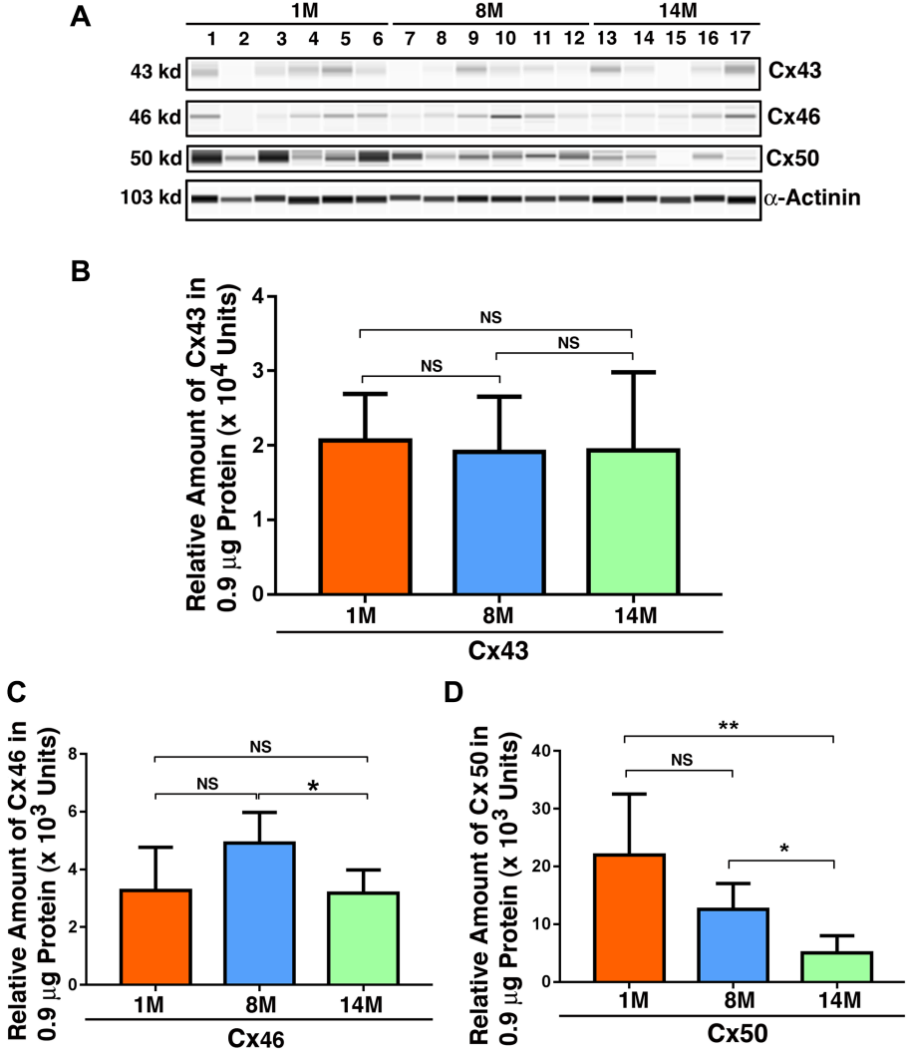
**Age-dependent changes of the connexins Cx43, Cx46 and Cx50 in mouse lens epithelial samples of different age groups as determined by the automated western immunoblot (AWI) analyses.** AWI was performed on a Wes (ProteinSimple) as described recently [48, 56]. Briefly, each sample was loaded with 0.9 μg total protein and then analyzed with the Size Separation Master Kit and Split Buffer (12–230 kDa) according to the manufacturer’s standard instruction using indicated antibodies (for antibody information, see Experimental Procedures) with a dilution factor of 1:100 for Cx43, and 1:20 for Cx46 and Cx50. The Campass software (Protein Simple, version 4.1.5) was used to program the PeggySue-robot and for presentation (**A**) and quantification (**B**–**D**). Output western blot style data (**A**) were displayed with the best exposure determined by the software, and the quantification data (**B**–**D**) were displayed from the software-calculated average of seven exposures (1–512s). (**B**) Quantification results show age difference of Cx43. (**C**) Quantification results show age difference of Cx46. (**D**) Quantification results show age difference of Cx50. NS, not significant, ^*^p < 0.05, ^**^p < 0.01.

Since we observed that gap junction proteins displayed significant changes in the epithelial samples from normal transparent lens to cataract lenses and also in the fiber cells of normal human lenses of different ages, we next sought to confirm that aging is a major factor in connexin downregulation. To achieve this, we examined Cx43, Cx46, and Cx50 in the lenses of 1M, 8M, and 14M mice. As shown in Figure 6A, individual variations existed for all three connexin subunits in all age groups. However, when all mice in the same age group were combined, the epithelial cell level of Cx43 in mouse lenses of all three different age groups displayed no significant change (Figure 6B). By contrast, the level of Cx46 in mouse lenses when comparing 1M with 8M exhibited some upregulation over this period. This was restored to 1M level between 8M and 14M (Figure 6C), a pattern similar to the patients from 60s to 70s, and also from 70s to 80s (Figure 2). Different from Cx43 and Cx46, Cx50 displayed steady downregulation from 1M to 8M, and also from 8M to 14M (Figure 6D), a situation similar to that found in patients from the 50s group to the 80s group (Figure 3).

### The capsular epithelia of mouse lenses have higher levels of Cx43 and Cx50 than Cx46

**Figure 7:**
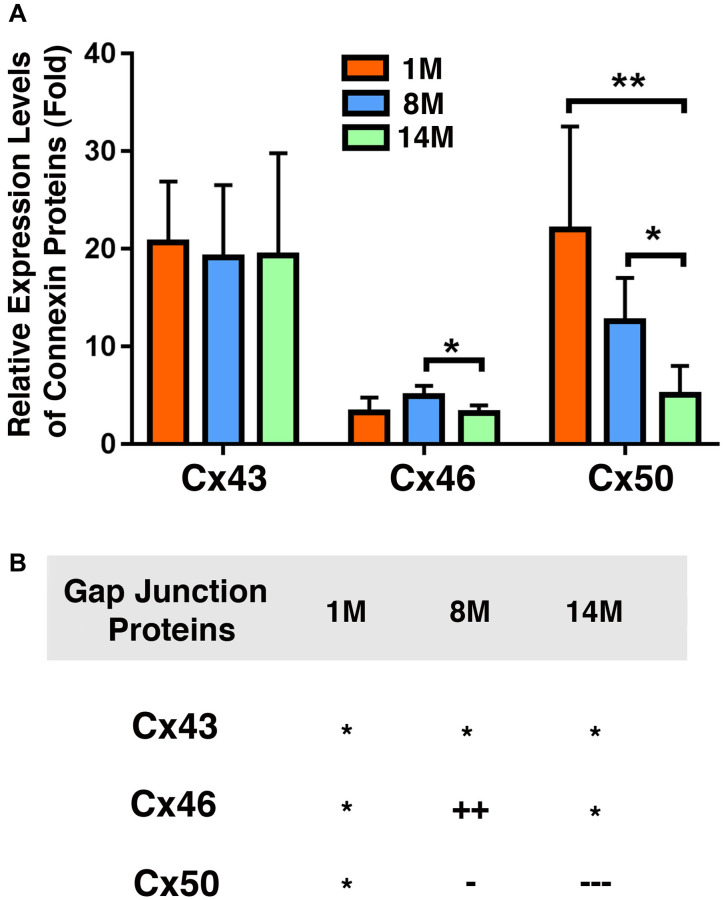
**Comparison of expression levels of the connexins Cx43, Cx46 and Cx50 in mouse lens epithelial samples of different age groups.** (**A**) Relative expression levels of Cx43, Cx46 and Cx50 in mouse lens epithelial samples of different age groups (1M, 8M and 14M). Each bar represents an average of five or more lens epithelial samples from five or more mice. (**B**) Summary of age-dependent changes of the connexins Cx43, Cx46 and Cx50 in mouse lenses of different age groups. The levels of the connexins Cx43, Cx46 or Cx50 from 1M old mice are used as references indicated by the star symbol ‘^*^’. +, ++, and +++ represent increases in protein levels between 0.1% and 24.99%, 25% and 50%, and >50%, respectively; −, −−, and −−− stands for decreases in protein levels between 0.1% and 24.99%, 25% and 50%, and >50%, respectively.

We next compared the relative levels of Cx43, Cx46, and Cx50 in mouse lens epithelial cells of different age groups. As shown in Figure 7A, Cx43 and Cx50 displayed similar levels in 1M mouse lenses, which was about six times higher than the level of Cx46 (Figure 7A). This is in sharp contrast with the ratio of Cx43 verse Cx46 and Cx50 in human lens, i.e., the Cx43 level is more than 90% higher than the levels of Cx46 and Cx50 (Figure 4). In addition, in human cataract lenses, the epithelial level of Cx46 is slightly higher than that of Cx50. During aging, Cx46 is upregulated from 1M to 8M. In contrast, Cx50 is once again steadily downregulated in mouse lens epithelia (Figure 7B). In summary, our results reveal that the distribution patterns of the three connexins are different in human and mouse lenses.

### Expression of Cx46 and Cx50 in mouse lens fiber cells of different age groups

**Figure 8:**
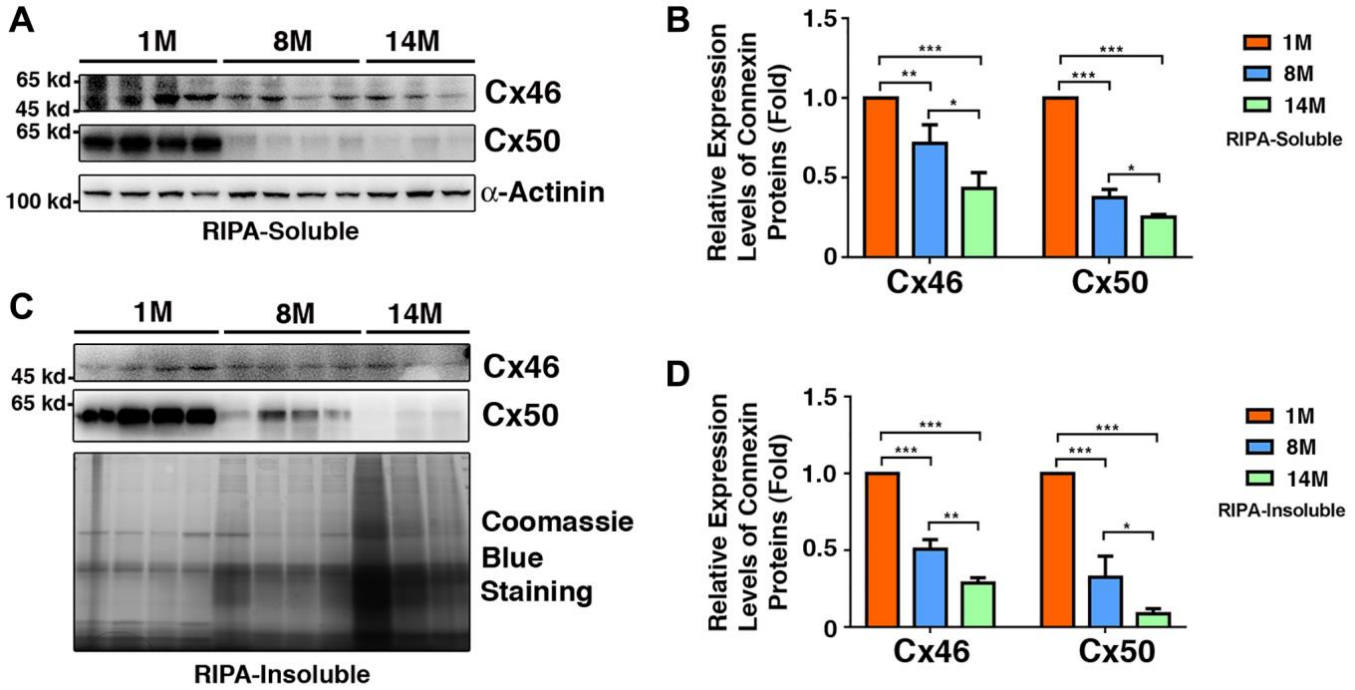
**Age-dependent changes of Cx46 and Cx50 in both RIPA-soluble and RIPA-insoluble fractions of lens fiber cells of different age groups of C57BL/6J mice as determined by regular western blot analysis.** (**A**) Western blot results of Cx46 and Cx50 in RIPA-soluble fraction of lens fibers of different age groups. Lanes 1–4 represent lens fiber samples from four 1M C57BL/6J mice, lanes 5–8 represent lens fiber samples from four 8M C57BL/6J mice, lanes 9–11 represent lens fiber samples from three 14M C57BL/6J mice. α-Actinin was showed as a loading control. (**B**) Quantification results show age-dependent changes of Cx46 and Cx50 in RIPA-soluble fraction of different age groups as determined in (**A**). (**C**) Western blot results of Cx46 and Cx50 in RIPA-insoluble fraction of lens fibers of different age groups. Lanes 1–4 represent lens fiber samples from four 1M C57BL/6J mice, lanes 5–8 represent lens fiber samples from four 8M C57BL/6J mice, lanes 9–11 represent lens fiber samples from three 14M C57BL/6J mice. α-Actinin was showed as a loading control. (**D**) Quantification results show age-dependent changes of the connexins Cx46 and Cx50 in RIPA-insoluble fraction of different age groups as determined in (**C**). ^*^p < 0.05, ^**^p < 0.01, ^***^p < 0.001.

Since Cx46 and Cx50 are major gap junction proteins in lens fiber cells, we next examined their age-dependent changes in the fiber cells of 1M to 14M mouse lenses. As shown in Figure 8A and 8B, in the RIPA-soluble fraction, both Cx46 and Cx50 displayed age-dependent downregulation, which is similar to previous results [50]. However, we noticed that the rate of downregulation was much faster for Cx50 than for Cx46, a situation similar to that found in human lens fiber cells (Figure 5). In the lens of 8M C57 mice, Cx46 displayed a 27% downregulation, and Cx50 exhibited a 68% downregulation. By 14M, Cx46 was downregulated by almost 50%, and Cx50 was decreased by more than 70%. Knowing that connexins are membrane proteins, we also examined the relative amount of both Cx46 and Cx50 in the RIPA-insoluble fraction. As shown in Figure 8C and 8D, both Cx46 and Cx50 also displayed age-dependent downregulation. In slight contrast to the soluble fraction, in the 8M fiber cells of mouse lenses, Cx46 was downregulated by 50%; Cx50, on the other hand, was downregulated by 64%. After 14M, about 27% of the Cx46 remained in this fraction. By contrast, less than 10% of the Cx50 was left.

To determine if the age-dependent downregulation of Cx46 and Cx50 depends on genetic background, we examined the age-dependent changes in Cx46 and Cx50 in the fiber cells of 2-month-old (2M) and 12-month-old (12M) lenses from S129 mice. As shown in Supplementary Figure 1, both Cx46 and Cx50 displayed age-dependent downregulation. Similar to C57 mice, the age-dependent downregulation of Cx46 was also slower than that of Cx50 (Supplementary Figure 1A). After 12M, Cx46 was downregulated by approximately 36% and Cx50 by approximately 68% (Supplementary Figure 1B).

### Oxidative stress differentially affects Expression of Cx43, Cx46, and Cx50 in mouse lenses

**Figure 9:**
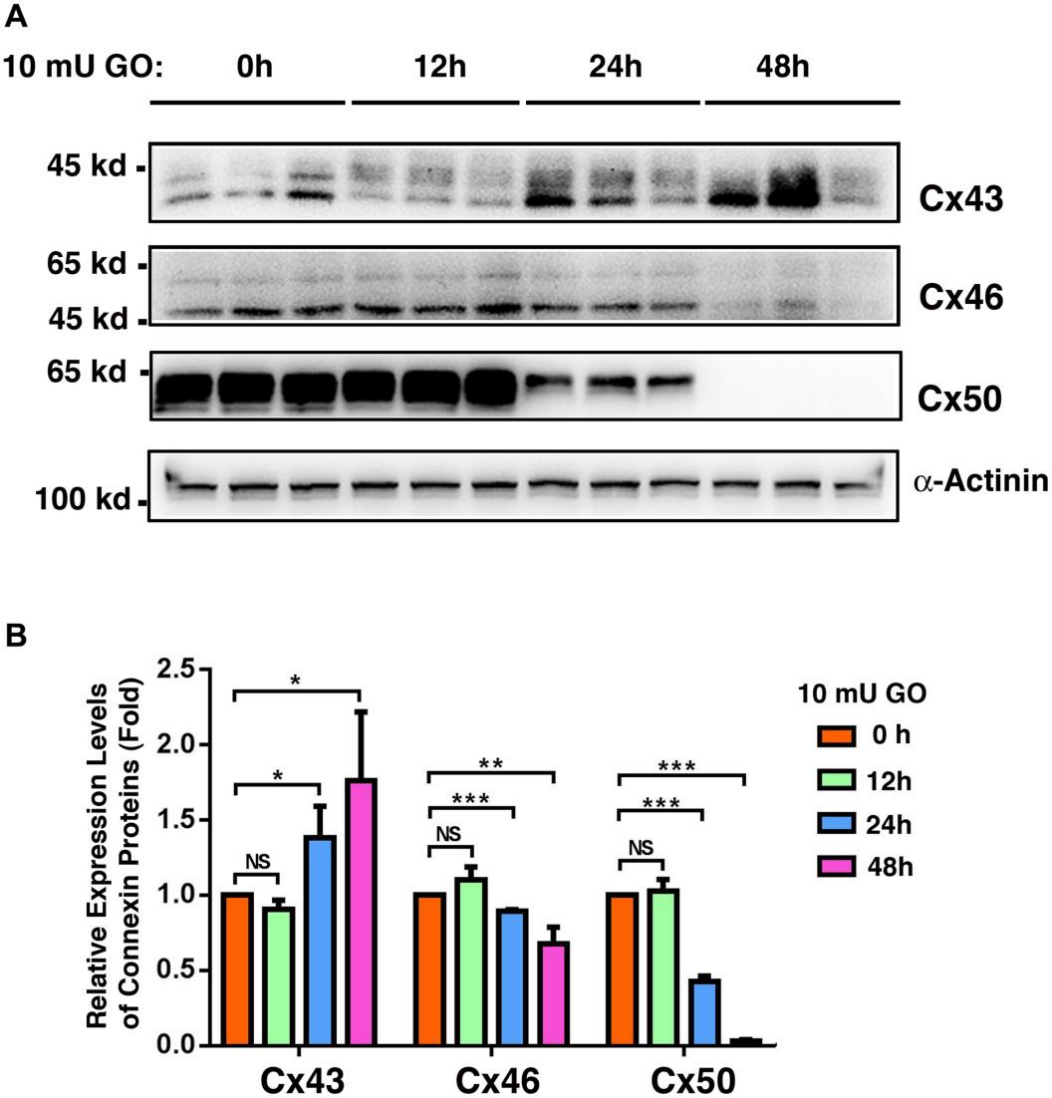
**Oxidative stress-induced changes of the connexins Cx43, Cx46 and Cx50 in the whole lenses of 4-week mice after treatment by 10 milli-units (mU) of glucose oxidase (GO) for different time.** Treatment of mouse lenses by 10 mU GO caused *in vitro* cataractogenesis in 24 and 48 hours (data not shown). (**A**) Western blot analysis of Cx46 and Cx50 in mouse lenses after treatment with 10 mU glucose oxidase for 0–48 h as indicated. α-Actinin was showed as a loading control. (**B**) Quantification of the western blot results in (**A**). NS, not significant, ^*^p < 0.05, ^**^p < 0.01, ^***^p < 0.001.

Oxidative stress is considered to be one of the major factors that cause aging [57–58]. Knowing that connexin proteins display clear age-dependent changes, we explored whether oxidative stress is capable of inducing changed expression patterns in the connexin proteins. In order to achieve this, transparent lenses isolated from 1M C57 mice were treated with 10 mU of glucose oxidase (GO), which causes the generation of hydrogen peroxide (Supplementary Figure 2A) and *in vitro* cataract development [48], and also leads to the downregulation of protein thiols as we previously demonstrated (Supplementary Figure 2B and also see ref. 48). Under this condition, Cx43 was slightly downregulated after 12 h of treatment, and subsequently became significantly upregulated after 24 h (a 37% increase) and 48 h of treatment (a 57% increase) (Figure 9A and 9B). By contrast, Cx46 and Cx50 were slightly upregulated after the first 12 h of treatment (Figure 9A and 9B). After 24 h of treatment, however, Cx46 and Cx50 displayed a 14% and 27% decrease, respectively. After 48 h of treatment, Cx46 and Cx50 exhibited a 55% and almost a 100% reduction, respectively (Figure 9A and 9B). The relative downregulation patterns of Cx46 and Cx50 induced by oxidative stress were similar to those induced by aging in fiber cells (Figure 8).

### Differential expression patterns of Cx43, Cx46, and Cx50 were detected in αA-/- and αB-/- knockout mouse lenses

**Figure 10:**
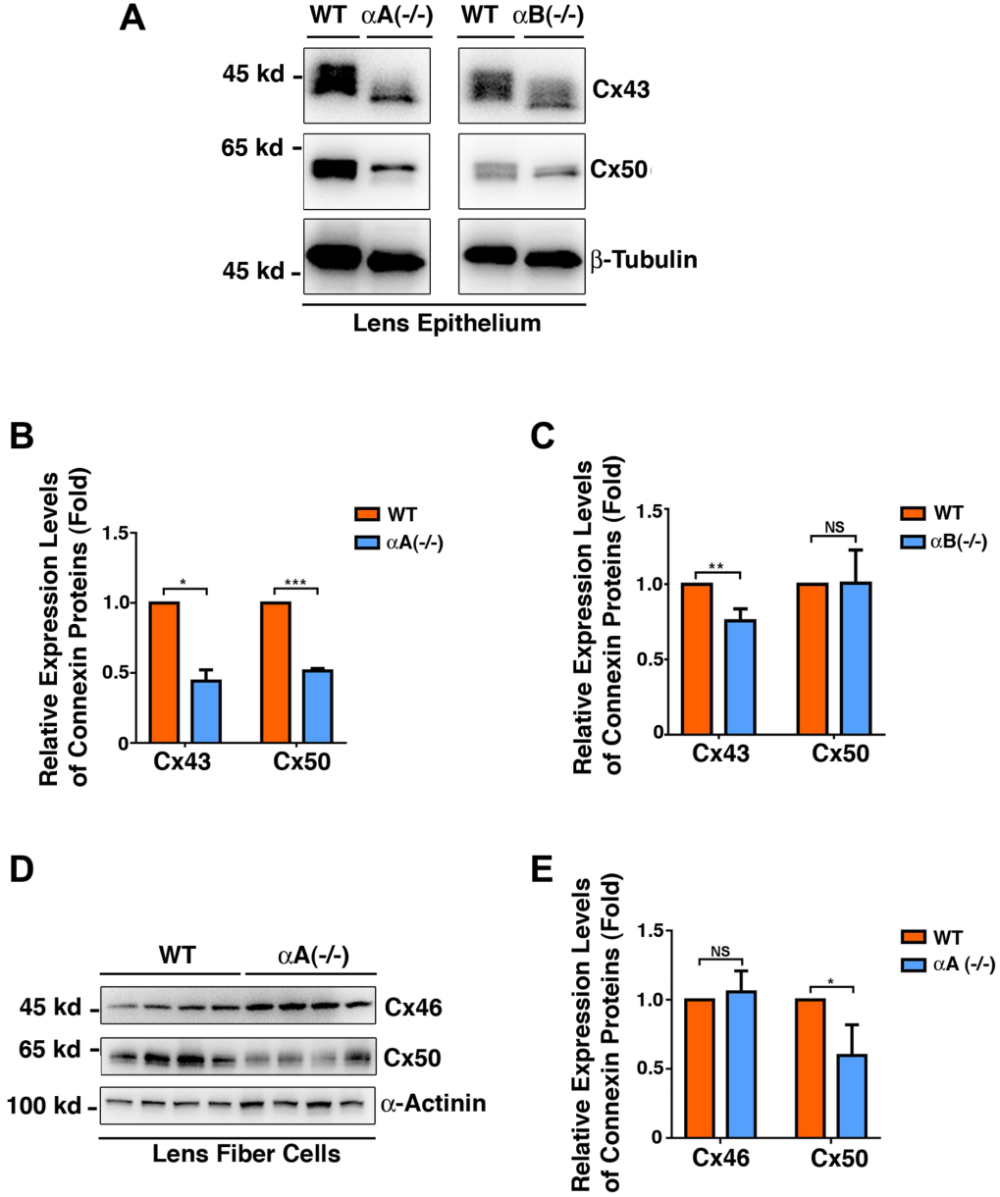
**Differential expression patterns of the connexins Cx43, Cx46 and Cx50 in the epithelial cells of the αA-/- and αB-/- mouse lenses (Figure 10A–10C), and in the fiber cells of the αA-/- mouse lenses (Figure 10D–10E).** (**A**) Western blot analysis of Cx43 and Cx50 in the lens epithelial cells of the αA-/- and αB-/- mice. β-Tubulin was showed as a loading control. (**B**) & (**C**) Quantification of the western blot results in (**A**). (**D**) Western blot analysis of Cx43 and Cx50 in the lens fiber cells of the αA-/- mice. α-Actinin was showed as a loading control. (**E**) Quantification of the western blot results in (**D**). NS, not significant, ^*^p < 0.05, ^**^p < 0.01, ^***^p < 0.001.

Since α-crystallins act as molecular chaperones and have been shown to protect other proteins of different types [59–64], we next examined if the expression patterns of the three connexin proteins were altered in αA-/- and αB-/- knockout mouse lenses. As shown in Figure 10A, 10B and 10C, in the lens epithelial cells, lack of αA-crystallin caused significant downregulation of Cx43 and Cx50. In contrast, lack of αB-crystallin only affected the stability of Cx43 but not Cx50. In the fiber cells, lack of αA-crystallin caused significant downregulation of Cx50 but displayed little effect on Cx46 (Figure 10D and 10E). Lack of αB-crystallin displayed no effect on the stability of either Cx46 or Cx50 (Supplementary Figure 3). Together, αA and αB-crystallins seem to have differential functions on the stability of the gap junction proteins.

## DISCUSSION

In the present study, we demonstrate the following: 1) From normal transparent lenses to cataract lenses, Cx43 is significantly upregulated, but both Cx46 and Cx50 are downregulated in the capsular epithelia; 2) In the fiber cells of normal human lenses over 50-year old, Cx46 and Cx50 are downregulated more than 90% with Cx50 decreasing much faster than Cx46; 3) Among different groups of cataract patients, the Cx43 level in the capsular epithelia is approximately nine times that of Cx46 and Cx50; 4) All three types of connexins display some gender difference from 70s to 80s, with the male connexin levels being higher than those in females; 5) Both Cx43 and Cx46 remain stable from 50s to 60s, and then display some upregulation from 60s to 70s; they then become significantly downregulated from 70s to 80s. By contrast, Cx50 displays steady downregulation from 50s to 80s; 6) During mouse lens aging, the Cx43 level remains unchanged in the epithelia from 1M to 14M, and Cx46 remains slightly upregulated from 1M to 8M but becomes downregulated from 8M to 14M. By contrast, Cx50 displays similar patterns of change to those found in the cataract patients of different age groups; 7) In mouse lens fiber cells, both Cx46 and Cx50 are downregulated from 1M to 8M and 14M. Moreover, the age-dependent decrease in Cx50 is much faster than that of Cx46, a pattern similar to that found in the fiber cells of normal human lenses; 8) Oxidative stress induces contrast expression patterns in the three connexins. While Cx43 is significantly upregulated, both Cx46 and Cx50 are sharply downregulated, with Cx50 decreasing much faster than Cx46; 9) αA and αB-crystallins seem to have differential functions on the stability of the gap junction proteins. In summary, for the first time, our results determine the differentially changing patterns of major subunits of lens gap junction proteins in normal human lenses, from transparent human lenses to cataractous lenses, and in different groups of cataract patients. We also extended the studies on the changing patterns of three connexins in aging mice. Together, our results for the first time reveal that downregulation of Cx46 and Cx50 appears to be the main reason for the diminished coupling conductance of the aged lens besides the age-dependent truncations at the defined sites [19–20], and that the aging-dependent loss of Cx46 and Cx50 promotes senile cataractogenesis.

### Human and mouse lenses have different distribution patterns of connexins proteins

Gap junction proteins play an important role in lens homeostasis and transparency [3–5, 11–25, 65–67]. They are responsible for transporting small molecular nutrients and metabolites [11–18, 24–25]. More recently, Liu et al. [21] elegantly demonstrated that these gap junction proteins are also capable of delivering antioxidants and maintaining the redox status of the ocular lens. Three GAP junction proteins: Cx43, Cx46, and Cx50, have been identified in the ocular lens [5]. It was revealed that mouse lens Cx43 is mainly expressed in the lens epithelial cells [6]. On the other hand, Cx46 is mainly expressed in the fiber cells [7]. In contrast to Cx43 and Cx46, Cx50 is expressed in both lens epithelial and fiber cells [7]. Our present study confirmed these results (Figures 6–9). However, our study of the three gap junction proteins in both transparent and cataractous human lenses revealed different distribution patterns; i.e., while Cx43 is indeed still the major isoform in the epithelial cells, both Cx46 and Cx50 are expressed in the lens epithelial cells of both transparent and cataractous lenses (Figures 2 to 4). As a matter of fact, in the transparent lenses of the three 60-year-old individuals, levels of Cx46 (1.8 × 10^4^ units, Figure 2C) were found to be more than 50% higher than those of Cx50 (7 × 10^3^ units, Figure 3C). In the cataractous lenses of the 70s group, levels of Cx46 (1.3 × 10^4^ units, Figure 4) were also approximately 50% higher than those of Cx50 (6.5 × 10^3^ units, Figure 4). In the 50s, 60s, and 80s age groups, the levels of Cx46 were also higher than those of Cx50 (Figure 4). Thus, in human lenses, both Cx46 and Cx50 are expressed in the lens epithelial cells and lens fiber cells. In addition, the expression patterns of Cx43, Cx46, and Cx50 in human lenses are different from those in mouse lenses in terms of the ratio between Cx43 and Cx46/Cx50. In human cataractous lenses, Cx43 is over nine times more abundant than Cx46 and Cx50 (Figure 4). By contrast, in the epithelial cells of mouse lenses, Cx43 is about five times more abundant than Cx46, but about equal to Cx50 in 1M mice (Figure 7). Thus, the distribution patterns of the three connexin subunits are different in human and mouse lenses. Regarding the localizations of Cx46 and Cx50 in the lens fiber cells, previous studies using two-photoflash photolysis reveal differences in both spatial distributions and age-dependent density changes [21–22].

### Molecular signature for senile cataract derived from analysis of connexin protein dynamics

The age-dependent changes in gap junction protein truncations have been explored in four pairs of human lenses aged 19, 22, 55 and 62 [19]. It was found that the truncations levels in the N-terminal domains and the cytoplasmic loops of Cx46 (G2, Q127, D128) and Cx50 (Q117, G119, N121, G122, G123, D125, Q126, G127) increased dramatically from outer cortex to nucleus. These truncations cause reduction in lens coupling conductance [19]. Cleavages of these two connexin proteins were mediated by the enzymatic action of calpain and other proteases or non-enzymatical reaction. Truncation-caused decrease in coupling conductance led to changes in transport of ions and important metabolites in the lens, for example, antioxidant cannot diffuse from the outer cortex to the fiber cells, resulting change in the glutathione redox state in the inner lens [25]. In addition, caspase-1/-3-mediated truncations at the C-terminal (Glu-368 and Asp-379) of chicken lens Cx50 also cause decrease in gap junction coupling [68]. Consistent with this observation, it was found that exogenously truncated cDNAs coding for Cx50 (Cx50tr290) and expressed in *Xenopus* oocytes exhibited an 86% to 89% reduction in mean macroscopic conductance compared with full length Cx50 cDNA injected. Thus, age dependent truncations of gap junction proteins act as one of the reasons for the reduction of the coupling conductance.

In the present study, our results reveal that from transparent lenses to cataractous lenses, Cx43 is upregulated by almost 50% (Figure 1). By contrast, Cx46 and Cx50 are downregulated by over 50%. Such a changing pattern suggests that loss of Cx46 and Cx50 functions may be compensated by Cx43. This is supported by an *in vitro* cataract model induced by glucose oxidase (GO). During treatment of mouse lenses with 10 mU GO, Cx43 was significantly upregulated after 24h and 48h of treatment (Figure 9). By contrast, Cx46 and Cx50 were significantly downregulated at the time when Cx43 became upregulated after 24 h and 48 h of treatment (Figure 9).

In the epithelial cells of transparent mouse lenses, Cx43 did not show significant changes during aging from 1M to 8M and 14M. Similarly, Cx46 was slightly upregulated from 1M to 8M and later returned to a level similar to that seen at 1M. In contrast to Cx43 and Cx46, Cx50 was steadily downregulated (Figure 7). In the fiber cells of mouse lenses from 1M to 8M, both Cx46 and Cx50 were downregulated (Figure 8). This downregulation pattern continued for Cx46 and Cx50 in the fiber cells of mouse lenses from 8M to 14M. Our results are consistent with a previous study of aging mouse lenses between 11 weeks and 11 months [55]. We also noticed that during aging of mouse lenses, Cx46 downregulation was slower than that of Cx50. This differential downregulation pattern of Cx46 and Cx50 was also observed in S129 mouse lenses (Supplementary Figure 1), in the fiber cells of normal human lenses, and in lens epithelial cells of human cataractous lenses (Figure 4).

In summary, our results have determined the dynamic changing levels of Cx43, Cx46, and Cx50 in aging human and mouse lenses. Besides truncations, over 90% downregulation of Cx46 and Cx50 in the fiber cells of human lenses aged 50 and above appears to be the main reason for the diminished coupling conductance. Both site specific truncations [19–20] and age-dependent loss of Cx46 and Cx50 can act as molecular signature for human cataract and aging lenses. Age-dependent loss of Cx46 and Cx50 promotes senile cataractogenesis.

### Oxidative stress-induced changes of connexins proteins contribute to cataractogenesis

During aging, reactive oxygen species (ROS) derived from the action of various oxidases, such as nicotinamide adenine dinucleotide phosphate (NADPH) oxidase, glucose oxidase, and lipoxygenase, can damage DNA, proteins, and membrane lipids [57]. The accumulation of oxidative stress-induced damages in these different macromolecules causes age-associated functional loss in different tissues and organs [58].

The cellular ROS components include superoxide anion (O_2_●), hydroxyl ion (OH●), and hydrogen peroxide (H_2_O_2_) [58]. Although H_2_O_2_ is not a free radical, through the Fenton or Haber–Weiss reaction, it can generate hydroxyl radicals that are extremely reactive, causing damage to proteins in the cytoplasm and phospholipids in the cellular membrane [57–58]. Several lines of evidence support the hypothesis that oxidative stress acts as one of the initiating factors in the formation of cataracts: an essential aging disease that causes blindness in developing countries [38]. First, in the human eye, the level of H_2_O_2_ is found to be elevated in the aqueous humor, i.e., from less than 25 μM in a normal lens to more than 50 μM in cataract patients [69]. Second, oxidative stress has been shown to damage DNA [49], proteins such as structural proteins in the lens [50], ion transporters [39–40], enzymes [51–53] and gap junction proteins [19–20, 68]. In this regard, hydrogen peroxide has been shown to induce Cx50 hemichannels open in lens fiber cells, which can mediate transport of reductant glutathione into fiber cells [70]. Two dominant mutants in Cx50, Cx50P88S and Cx50H156N, inhibit transport through Cx50 hemichannels. These mutants thus can augment the effects of hydrogen peroxide in promoting apoptosis of lens fiber cells [70]. In addition, it has been shown that oxidative stress derived from 4-hydroxynonenal (4-HNE) can inhibit Cx46 hemichannels’ function through its carbonylation [71].

In the present study, we demonstrated that oxidative stress generated from glucose oxidase causes significant upregulation of Cx43 and downregulation of Cx46 and Cx50 (Figure 9). Such differential changes likely lead to the observed restrictions of the intercellular communications in the oxidative stress-damaged lens as well as aging lenses [72–74]. Oxidative stress-induced changes in the post-translational modifications, such as phosphorylation, can also alter the conductance of the gap junction proteins [75–76]. It has been shown that oxidative stress derived from the knockout (KO) of glutathione peroxide-1 (GPX-1) causes both degradation of Cx46 and Cx50 and loss of the coupling conductance in the GPX-1 KO mice [77]. Oxidative stress-induced damages to various molecules eventually trigger apoptosis of the lens epithelial cells [41–42, 70, 78–79], leaving the underlying fiber cells unprotected, and leading to cataract formation [41–42, 70, 78–79].

### αA- and αB-crystallins have differential functions on the stability of gap junction proteins

α-Crystallins are major lens structure proteins and consist of two polypeptides, αA- and αB-crystallins that share 55% amino acid sequence identity [80–81]. The two 20-kDa subunits form soluble aggregates with an average molecular mass of 600–800 kDa and can be isolated from lens fiber cells as a heteroaggregate containing αA- and αB-peptides in a ratio of 3 to 1 [80–81]. Besides their structural role, α-crystallins are important chaperones in the ocular lens [59–64]. The *in vitro* experiments have demonstrated that they can prevent denaturation of a large variety of proteins from enzymes, lens crystallins, to cytoskeletons [59–64]. Here, we demonstrated that in αA-/-mice, both Cx43 and Cx50 are significantly downregulated in lens epithelial cells, and Cx50 was also downregulated in the fiber cells (Figure 10). In contrast, only Cx43 was downregulated in αB-/-mice (Figure 10). Thus, αA and αB-crystallins display differential functions in regulating the stability of lens gap junctions. The effects of αA and αB-crystallins on gap junction proteins appear to be indirect since our Co-IP experiments failed to detect the direct interaction between αA and αB-crystallins and three gap junction proteins (data not shown).

## MATERIALS AND METHODS

### Animals

The study was performed using the following mice: 1-month (1M), 8-month (8M), and 14-month (14M) old C57BL/6J mice; and 2-month (2M) as well as 12-month (12M)-old S129 mice; and 1-month old αA-/- and αB-/- mice. Mice were housed in standard cages in a specific pathogen-free facility of Sun Yat-Sen University. The room was maintained on a 12 h light-dark cycle at a constant temperature of 25°C and 50% humidity, and the animals were fed with commercial laboratory food and sterilized water. The animal protocol for mouse usage was approved by the IACUC of Zhongshan Ophthalmic Center of Sun Yat-Sen University.

### Lens organ culture

The C57BL/6J and S129 mice of different ages were sacrificed by CO_2_ inhalation. The eyeballs were removed and the lenses were carefully dissected using a posterior approach [41–42]. Dissected lenses were placed in a 10-cm dish containing 20 ml of meidium199, then incubated at 37°C with a 5% CO_2_ gas phase for 12h. The medium 199 was prepared with ion-exchange double-distilled water and supplemented with 26mM NaHCO_3_, with a pH adjusted to 7.2, then it was sterilized by filtration through a 0.22um filter. After 12 h culture, transparent lenses were selected for further experimentation.

### Glucose oxidase (GO) treatment

For each sample, three transparent lenses were transferred into a 6-cm petri dish containing 7 ml of medium 199 supplemented with 10 mU glucose oxidase (GO) [48, 82], which continuously generates an average of 100 μM of cytotoxic H_2_O_2_ in a 48 h period.

### Measurement of hydrogen peroxide and free thiol levels

The free thiol content was determined with a fluorometric thiol quantitation kit (Sigma-Aldrich Corp., #MAK151) according to the manufactory’s instruction. Briefly, mouse lenses were treated with 10 mU GO for 0 to 48 h. After GO treatment, the lens was washed with phosphate buffered-saline (PBS) three times, homogenized in 250 μl of lysis buffer, and 25 μl of the extracted proteins were used for each assay reaction. Hydrogen peroxide in the culture media was measured as described before [41].

### Collection of lens capsular epithelial samples

The collection of human capsular epithelia from cataract lenses of different age groups was approved by the Institutional Review Board of the Zhongshan Ophthalmic Center (ZOC). Informed consent was obtained from each of the cataract patients. For senile cataractous samples, the lens capsules from cataract patients were collected at surgery by the physicians in Zhongshan Ophthalmic Center of Sun Yat-Sen University. According to the patient age, capsular samples from 50 to 59 years old were grouped together and labeled as 50s (Supplementary Table 2); those from 60 to 69 years old were grouped together and labeled as 60s (Supplementary Table 3); those from 70 to 79 years old were grouped together and labeled as 70s (Supplementary Table 4), and those from 80 to 89 years old were grouped together and labeled as 80s (Supplementary Table 5). As control, the capsular samples from the lenses of human donors (Supplementary Table 1, one 45-year old female, and three male individuals aged 61, 64 and 65), and adult mice of different ages as described in the section above were dissected in the laboratory. Normal fiber cells were isolated from six human lenses [see Supplementary Table 1 for the 6 donors, one 7-month (7M) female, one 54-year old female, three male individuals aged 61, 64 and 65, and one 74-year old female].

### Total protein extraction and western blot analysis

Total proteins were extracted from cultured mouse lenses with RIPA buffer (50 mM Tris.HCl (pH7.4), 150 mM NaCl, 2 mM EDTA, 1% NP-40, 0.1% SDS, 1% sodium deoxycholate) and homogenized as previously described [48, 83–84]. For various capsular samples of lens epithelia, each capsular sample from an individual normal lens donor or cataract patient (see Supplementary Table 1 to Supplementary Table 5 for details), or from an individual mouse lens was transferred to an Eppendorf tube containing 50 μl RIPA buffer and homogenized on ice with an Eppendorf tube micropestle (Brinkmann Instruments Inc.). For each sample, the protein concentration was determined as previously described [48, 83–84]. For Automated Wes analysis, 0.9 μg of total proteins was used in each sample (see below for the method). For regular western blot analysis, 20, 50 or 150 μg of total proteins from lens fiber cells were separated by 10% SDS-PAGE gel and transferred into PVDF membranes. The protein blots were blocked with 5% non-fat milk in TBST (10 mM Tris HCl/pH8.0, 150 mM NaCl, 0.05% Tween-20) for 1 h at room temperature. Each membrane was then incubated with anti-Cx43 (rabbit polyclonal, 1:1000, CST, 3512S), anti-Cx46 (mouse monoclonal, 1:200, Santa Cruz Biotechnology, sc365394), anti-Cx50 (mouse monoclonal, 1:200, Santa Cruz Biotechnology, sc373801), or anti- α-actinin (rabbit polyclonal, 1:5000, Wuhan Proteintech, 11313-2-AP) antibodies at 4°C overnight with mild shaking. After washing three times for 10 min each with TBST, each blot was incubated with the HRP-conjugated secondary antibody (anti-mouse and rabbit IgG from CST) diluted at 1:2000 in blocking solution at room temperature for 1h. The blots were visualized using a Tanon chemiluminescence system (China).

### Automated western immunoblotting

The automated western immunoblots were performed on a Wes (ProteinSimple) as previously described [48, 56]. Briefly, each sample was loaded with 0.9 μg total protein extracted from the capsular epithelium of individual normal human lens, or capsular epithelial sample of each cataractous lens at surgical operation, or each capsular epithelial sample from individual mouse lens of different age groups, and then analyzed with the Size Separation Master Kit and Split Buffer (12–230 kDa) according to the manufacturer’s standard instruction using the antibodies described above. The dilution factors were 1:500 for α-actinin; 1:100 for Cx43, and 1:20 for Cx46 and Cx50. The Campass software (Protein Simple, version 4.1.5) was used to program the Wes-robot and for presentation and quantification of the western blots. Output western blot style data were displayed with exposure time indicated or the best time as determined by the software. The quantification data were displayed from the software-calculated average of seven exposures (1–512 s).

### Statistical analysis

The results presented in the figures are representative of three or more independent repetitions. All data were analyzed with SPSS 17.0 software (SPSS Inc., Chicago, IL). One way analysis of variance (ANOVA) followed by the Tukey’s for multiple comparisons were used for statistical analyses. The *p* value < 0.05 was considered statistically significant. *, ** and *** represent *p* < 0.05, 0.01 and 0.001, respectively.

### Data availability statement

All data are available upon request. Raw data and processed data will be made available at Gene Expression Omnibus (https://www.ncbi.nlm.nih.gov) upon acceptance of the manuscript for publication.

## Supplementary Materials

Supplementary Figures

Supplementary Tables
