# Supplementary material for: Aging-dependent loss of GAP junction proteins Cx46 and Cx50 in the fiber cells of human and mouse lenses accounts for the diminished coupling conductance
Source: Aging (Albany NY). 2021 Jul 4;13(13):17568–91. doi: 10.18632/aging.203247 (PMC8312418; doi:10.18632/aging.203247)
Supplement: Supplementary Figures [file aging-13-203247-s001.pdf]

## SUPPLEMENTARY FIGURES

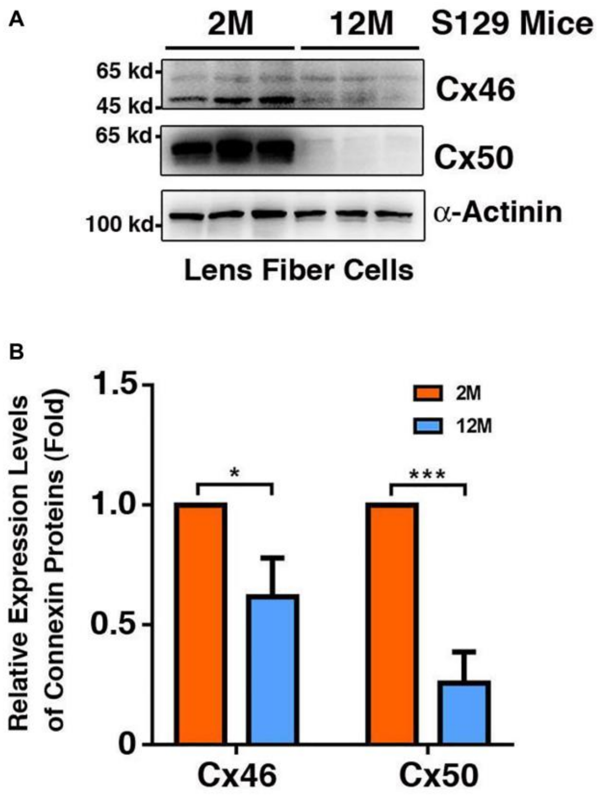


**Supplementary Figure 1. Age-dependent changes of the connexins Cx46 and Cx50 in lens fiber cells of different age groups of S129 mice as determined by regular western blot analysis.** (**A**) Western blot results of the connexins Cx46 and Cx50 in lens fiber cells of different age groups extracted with SDS buffer. Lanes 1–3 represent lens fiber samples from three 2M S129 mice, lanes 4–6 represent lens fiber samples from three 12M S129 mice. α-Actinin was showed as a loading control. (**B**) Quantification results show age-dependent changes of the connexins Cx46 and Cx50 in the mouse lens fiber cells of different age groups as determined in (**A**). 2M: 2-month, 12M: 12-Month; $^{*}p < 0.05$, $^{***}p < 0.001$.

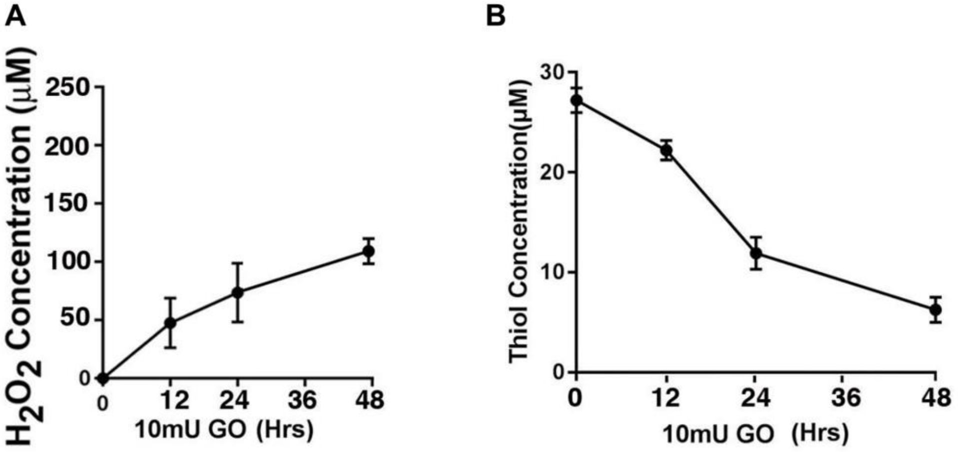


**Supplementary Figure 2.** (**A**) Dynamic $H_2O_2$ concentration generated from 10 milli-units (mU) of glucose oxidase (GO) in the M199 medium in which normal transparent mouse lenses were cultured for 0, 12, 24 and 48 hours in 10-cm culture dish with 30 ml medium. (**B**) Dynamic changes of free thiol levels in mouse lens under 10 mU GO treatment.

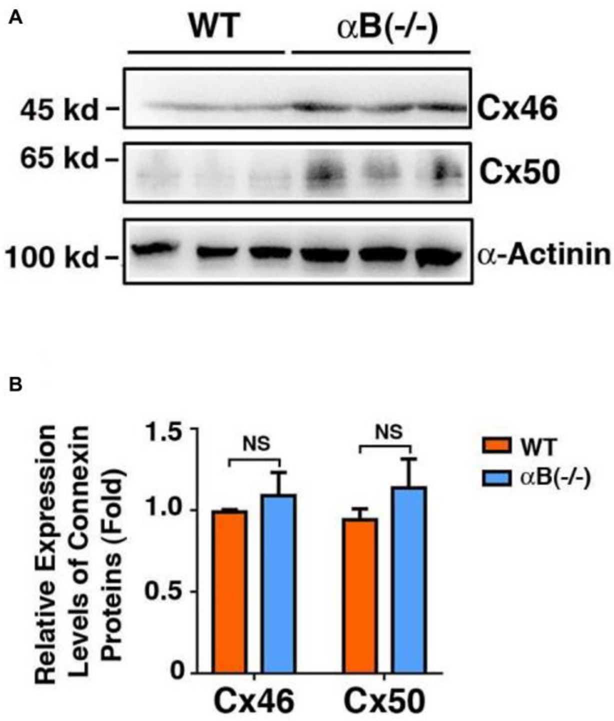


**Supplementary Figure 3. Expression Patterns of the connexins Cx46 and Cx50 in lens fiber cells of wild type (WT) and αB knockout (αB-/-) mice as determined by regular western blot analysis.** (**A**) Western blot results of the connexins Cx46 and Cx50 in the lens fiber cells of wild type (WT) and αB knockout (αB-/-) mice. (**B**) Quantification results show the connexins Cx46 and Cx50 in the lens fiber cells of wild type (WT) and αB knockout (αB-/-) mice as determined in (**A**). NS, not significant.
