# Supplementary material for: Aging-dependent loss of GAP junction proteins Cx46 and Cx50 in the fiber cells of human and mouse lenses accounts for the diminished coupling conductance
Source: Aging (Albany NY). 2021 Jul 4;13(13):17568–91. doi: 10.18632/aging.203247 (PMC8312418; doi:10.18632/aging.203247)
Supplement: Supplementary Tables [file aging-13-203247-s002.pdf]

SUPPLEMENTARY TABLES

Supplementary Table 1. Donor information of normal human lenses.

Number	Gender	Age	Lens tissue used
D1	Female	45	Lens Epithelium
D2	Male	65	Lens Epithelium and Fibers
D3	Male	64	Lens Epithelium and Fibers
D4	Male	61	Lens Epithelium and Fibers
D5	Female	7-month	Lens Fibers
D6	Female	54	Lens Fibers
D7	Female	74	Lens Fibers

Supplementary Table 2. Senile cataract patient information of 50s (50–59 years old).

Number	Gender	Age	Diagnosed subtype
P1	Male	50	Cortical cataract
P2	Male	53	Nuclear Cataract
P3	Male	54	Cortical Cataract
P4	Male	56	Nuclear Cataract
P5	Male	57	Nuclear Cataract
P6	Female	59	Nuclear Cataract
P7	Female	52	Nuclear Cataract
P8	Female	54	Nuclear Cataract
P9	Female	54	Nuclear Cataract
P10	Female	56	Cortical Cataract
P11	Female	56	Nuclear Cataract
P12	Female	57	Cortical Cataract

Supplementary Table 3. Senile cataract patient information of 60s (63–68 years old).

Number	Gender	Age	Diagnosed subtype
P1	Male	63	Nuclear Cataract
P2	Male	64	Nuclear Cataract
P3	Male	65	Cortical Cataract
P4	Male	66	Cortical Cataract
P5	Male	67	Nuclear Cataract
P6	Female	68	Cortical Cataract
P7	Female	64	Cortical Cataract
P8	Female	66	Cortical Cataract
P9	Female	66	Nuclear Cataract
P10	Female	66	Nuclear Cataract
P11	Female	66	Nuclear Cataract
P12	Female	67	Cortical Cataract

Supplementary Table 4. Senile cataract patient information of 70s (74–76 years old).

Number	Gender	Age	Diagnosed subtype
P1	Male	75	Cortical Cataract
P2	Male	75	Cortical Cataract
P3	Male	75	Nuclear Cataract
P4	Male	75	Nuclear Cataract
P5	Male	76	Nuclear Cataract
P6	Female	76	Nuclear Cataract
P7	Female	74	Nuclear Cataract
P8	Female	74	Nuclear Cataract
P9	Female	75	Cortical Cataract
P10	Female	76	Cortical Cataract
P11	Female	76	Nuclear Cataract
P12	Female	76	Nuclear Cataract

Supplementary Table 5. Senile cataract patient information of 80s (81–87 years old).

Number	Gender	Age	Diagnosed subtype
P1	Male	81	Cortical Cataract
P2	Male	82	Nuclear Cataract
P3	Male	85	Nuclear Cataract
P4	Male	85	Nuclear Cataract
P5	Male	85	Nuclear Cataract
P6	Female	86	Posterior Subcapsular Cataract
P7	Female	82	Nuclear Cataract
P8	Female	85	Nuclear Cataract
P9	Female	85	Posterior Subcapsular Cataract
P10	Female	85	Nuclear Cataract
P11	Female	85	Nuclear Cataract
P12	Female	87	Nuclear Cataract
